# Clinical trials in low‐resource settings: the perspectives of caregivers of paediatric participants from Uganda, Tanzania and Kenya

**DOI:** 10.1111/tmi.13281

**Published:** 2019-07-01

**Authors:** Machteld van den Berg, Bernhards Ogutu, Nelson K. Sewankambo, Sonja Merten, Nikola Biller‐Andorno, Marcel Tanner

**Affiliations:** ^1^ Institute of Biomedical Ethics and History of Medicine University of Zurich Zurich Switzerland; ^2^ Swiss Tropical and Public Health Institute Basel Switzerland; ^3^ University of Basel Basel Switzerland; ^4^ Kenya Medical Research Institute Nairobi Kenya; ^5^ CREATES Strathmore University Nairobi Kenya; ^6^ College of Health Sciences Makerere University Kampala Uganda

**Keywords:** low‐resource settings, clinical trials, vaccine development, complexity theory, caregivers, qualitative research, régions à faibles ressources, essais cliniques, développement de vaccins, théorie de la complexité, soignants, recherche qualitative

## Abstract

**Objectives:**

Vaccine clinical trials in low‐resource settings have unique challenges due to structural and financial inequities. Specifically, protecting participant and caregiver autonomy to participate in the research study can be a major challenge, so understanding the setting and contextual factors which influence the decision process is necessary. This study investigates the experience of caregivers consenting on behalf of paediatric participants in a malaria vaccine clinical trial where participation enables access to free, high‐quality medical care.

**Methods:**

We interviewed a total of 78 caregivers of paediatric participants previously enrolled in a phase II or III malaria vaccine clinical trial in Uganda, Tanzania and Kenya. Interviews were qualitative and analysed using a thematic framework analysis focusing on the embodied caregiver in the political, economic and social reality.

**Results:**

Caregivers of participants in this study made the decision to enrol their child based on economic, social and political factors that extended beyond the trial into the community and the home. The provision of health care was the dominant reason for participation. Respondents reported how social networks, rumours, hierarchal structures, financial constraints and family dynamics affected their experience with research.

**Conclusions:**

The provision of medical care was a powerful motivator for participation. Caregiver choice was limited by structural constraints and scarce financial resources. The decision to participate in research extended beyond individual consent and was embedded in community and domestic hierarchies. Future research should assess other contexts to determine how the choice to participate in research is affected when free medical care is offered.

## Abstract (fr)

**Objectifs:**

Les essais cliniques de vaccins dans les régions à faibles ressources présentent des défis uniques en raison d'iniquités structurelles et financières. En particulier, la protection de l'autonomie des participants et des soignants dans la participation à l’étude de recherche peut être un défi majeur. Il est donc nécessaire de comprendre le contexte et les facteurs contextuels qui influencent le processus de décision. Cette étude examine l'expérience de soignants consentants au nom de participants pédiatriques à un essai clinique de vaccin antipaludique où la participation permet l'accès à des soins médicaux gratuits et de haute qualité.

**Méthodes:**

Nous avons interviewé un total de 78 soignants de participants pédiatriques qui avaient participé à un essai clinique de vaccin antipaludique de phase II ou III en Ouganda, en Tanzanie et au Kenya. Les entretiens ont été qualitatifs et analysés à l'aide d'une analyse de cadre thématique centrée sur le soignant, en tenant compte de la réalité politique, économique et sociale.

**Résultats:**

Les soignants des participants à cette étude ont pris la décision d'inscrire leur enfant sur base de facteurs économiques, sociaux et politiques qui s’étendaient au‐delà de l'essai dans la communauté et à la maison. La fourniture de soins de santé était la principale raison de la participation. Les répondants ont rapporté comment les réseaux sociaux, les rumeurs, les structures hiérarchiques, les contraintes financières et la dynamique familiale ont affecté leur expérience avec la recherche.

**Conclusions:**

La fourniture de soins médicaux était un puissant facteur de motivation pour la participation. Le choix du soignant était limité par des contraintes structurelles et les ressources financières limitées. La décision de participer à la recherche allait au‐delà du consentement individuel et s'inscrivait dans les hiérarchies communautaires et domestiques. Les recherches futures devraient évaluer d'autres contextes afin de déterminer comment le choix de participer à la recherche est affecté par la gratuité des soins médicaux.

## Introduction

Vaccines play a major role in public health and their development is dependent on clinical trial testing in human populations. Transnational clinical trials operate through collaborative partnerships that involve a wide array of stakeholders and participants from varying sociocultural backgrounds. Each stakeholder enters into clinical trial research with varying degrees of inequity linked to its role in the clinical trial and resource context 1, 2. This inequity is of particular relevance for vaccine research operating in low‐resource settings due to the discrepancy in resources between the trial centre and the research site in which it operates, which has implications for the choice to participate in research 3.

Phase II and phase III vaccine clinical trials establish the safety of a vaccine and determine its efficacy 4. These trials involve large groups of participants who are living in the region where the disease targeted by the vaccine is endemic. Due to the operation of phase II and III trials in human communities, the social structures in the clinical trial site are highly relevant to the clinical trial design. To maximise the effectiveness of vaccines currently in clinical trial development, research needs to be sensitive to the social systems within the context in which they are operating 1, 2. To gain a substantive understanding of the social system in the clinical trial site, communities must be engaged and voices of participants and their families heard 3, 5, 6, 7, 8. This engagement process provides insight into the decision‐making structures around trial participation, communication needs and the interests of the community in the trial context.

This study investigates the community context, communication needs and decision‐making processes of the caregivers of participants in a phase II and phase III paediatric malaria vaccine clinical trial. Each of these trials operated in low‐resource settings in multiple African countries. The phase II vaccine trial involved GMZ2 malaria vaccine and was conducted at five clinical trial centres in four African countries 9. The phase III trial involved RTS,S malaria vaccine and was conducted at eleven clinical trial centres in seven countries 10. Operating across social systems, these transnational clinical trials provide insight into the impact that vaccine clinical trials have on the local population while adhering to standardised clinical trial protocols. Local systems and cultures influence decision‐making in clinical trial research and mapping the country‐specific context supports successful transnational research for development 11, 12, 13. This study takes these clinical trials as case studies to map the country‐specific context and shed light on the caregiver and community experiences in clinical research in low‐resource settings.

The phase III RTS,S clinical trial investigated here has led to the regulatory registration and the roll‐out of the RTS,S vaccine in a phase IV study, making it the first licenced malaria vaccine. These phase IV studies will take place in three different countries, including the Kenyan research centres investigated here 14. This makes the experiential understanding of the research participants, their caregivers and communities in the context of the clinical trial centre even more pertinent.

While community engagement has been recognised as necessary in ethical transnational research, there is no clear consensus as to its definitive application in different community contexts 15. Context may influence the ways in which benefits and risks are perceived by the participants, particularly in settings with large resource inequities 16, 17. Using knowledge of the health and social structures to inform community engagement practice is a critical component of designing research studies appropriately 18. Researchers conducting clinical trial studies in low‐resource settings can integrate the findings of this study to protect participant autonomy through integrating them into the communication of trial procedures.

## Methods

In order to better understand the experiences and decision‐making processes of caregivers during the paediatric malaria vaccine clinical trial, we conducted a series of in‐depth interviews between March 2017 and March 2018 with caregivers of children who participated in a malaria clinical trial.

### Sample population

Interviews were held across four clinical trial sites in Uganda (Iganga), Kenya (Siaya and Kombewa) and Tanzania (Bagamoyo) with caregivers of participants. We used purposive sampling to recruit respondents and in the majority of the cases the mother was the primary caregiver. Participants were selected based on having had a child enrolled in a paediatric malaria vaccine clinical trial. Interviews were conducted until saturation was reached, were semi‐structured and held in the home of the respondent.

### Trial

The RTS,S phase III malaria vaccine trial took place between March 2009 and January 2014 in seven African countries (Burkina Faso, Gabon, Ghana, Kenya, Malawi, Mozambique, United Republic of Tanzania) and spanned 11 clinical trial centres 10. The GMZ2 phase IIb malaria vaccine trial took place between April 2010 and July 2012 in four African countries (Burkina Faso (2), Ghana (1), Uganda (1), Gabon (1)) and spanned five clinical trial centres 9.

### Study design

This was a qualitative study that used in‐depth interviews to capture the perspective of the caregiver who had a child enrolled in a malaria vaccine clinical trial. The fieldwork consisted of a scoping trip to the research sites to introduce the study, recruit participants and meet with community leaders. Field visits took place in March 2017 (the scoping visit), and the interviews were conducted between May 2017 and March 2018 with the help of local research assistants. The research assistants from Tanzania were fluent in Swahili (one female and one male). In Uganda, all three female research assistants were fluent in Luganda and conversational in the related Lusoga language of the community investigated. In Kenya, all three research assistants (one male and two female) were fluent in Dholuo. None of the research assistants lived in the community investigated, all were fluent in English and had post‐secondary education. Interviews were semi‐structured and had a focused discussion on the vaccine trial, leaving room to explore concepts as they emerged, such as community dimensions and domestic relationships in the context of the trial. Interviews began by asking open questions and were then funnelled into more specific questions about the respondent's views and experiences within the health system, interaction with researchers, and challenges faced in the community. The interviews were recorded with the informed consent of the respondent and conducted in the local language. They were then transcribed verbatim and translated into English by the research assistant. The interview guide was first piloted in each country and then changed and developed throughout to best explore unanticipated replies as they emerged.

### Ethics

The study protocol, informed consent forms and interview guide were reviewed and approved by the following bodies: in Tanzania, National Health Research Ethics Review Committee for the National Institute for Medical Council (NIMR), Ifakara Health Institute IRB (IHI‐IRB), Tanzania Commission for Science and Technology (COSTECH); in Uganda, Uganda Council for Science and Technology (UNCST), the Makerere University School of Biomedical Sciences Higher Degrees Research and Ethics Committee (SBS‐HDREC); in Kenya: Strathmore University IRB (SU‐IRB).

### Analysis

**Figure 1:**
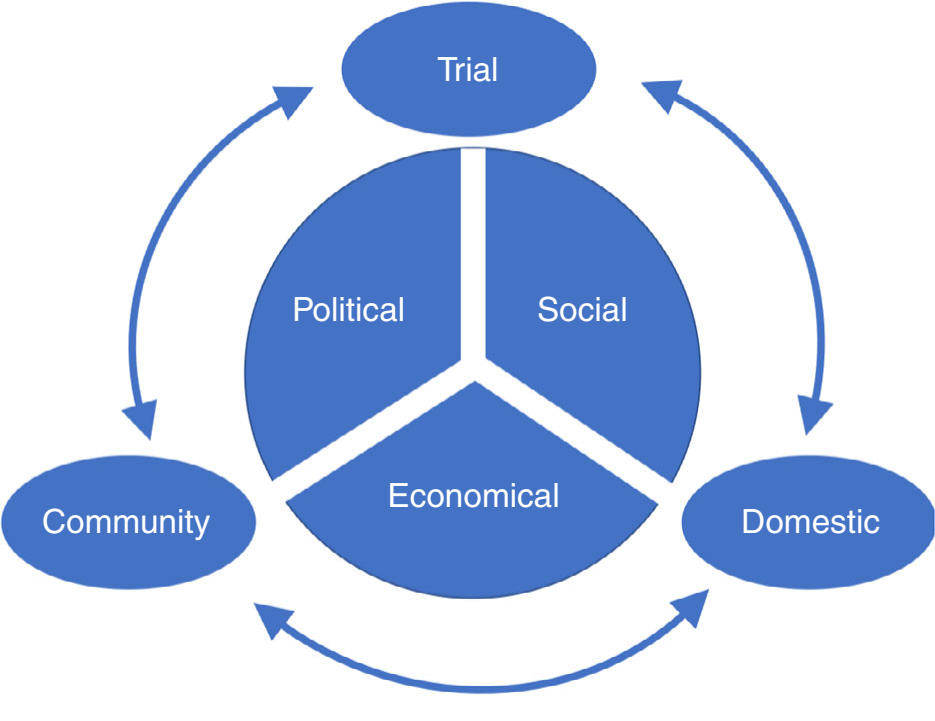
Framework for analysis outlining the interplay between community, domestic and trial contexts that the respondent inhabits and the economic, political and social realities of the embodied respondent. [Colour figure can be viewed at http://wileyonlinelibrary.com]

The analysis was based on the approach described by Strauss and Corbin (1998) 19. First, a detailed line‐by‐line microanalysis was conducted to identify categories in the data, followed by an exploration of the categories, their properties and the relationships between them. This was discussed between the first author of this paper and the local bilingual research team to ensure the accuracy of the analytical process. Categories were defined into main themes as illustrated in Figure 1. The themes were then integrated into a framework to define the scope of the analysis and are presented in the results.

## Results

Of the 78 interviews, 23 were with parents of children enrolled in the GMZ2 Phase IIb trial in Iganga, Uganda. The remaining 55 in‐depth interviews were with parents of children enrolled in the RTS,S phase III study across three sites in Bagamoyo, Tanzania (*n* = 18), Kombewa, Kenya (*n* = 20) and Siaya, Kenya (*n* = 17). Interviews lasted around 31 min on average, with the longest being 56 min and the shortest being 19 min. Seven interviews could not be included in the time calculation due to logistical limitations of the recordings.

The respondents made it explicit that they inhabit multifaceted realities falling under clinical trial, community and domestic contexts. Each of these themes is shaped and embedded in their economic, social and political reality (Table 1).

**Table 1:** Respondents reported their experience of the trial, community and domestic context. The dominant themes that arose during the interviews are displayed

Setting	Dominant themes
Trial context	Social networks reported on high‐quality care Trial centre vs. government facility medical care Social skills of trial doctors Outlier: trial led to rumours and was impacted by them
Community context	Community valued health care Reassured by friends about participation Health benefits should be for the whole community Trial improved conditions in the community Local leadership influences community acceptance Outlier: Rumours impacted social life
Domestic context	Individual context leads to trial enrolment Valued health care and improved condition of child Fathers influenced enrolment, consent and withdrawal Explicit reality of sick child in the home Outlier: Trial interfered with domestic harmony

### Trial context

Respondents frequently began by explaining their trial experiences and defined the role the trial played in their lives. They did not view the clinical trial as detached from their lives, instead participation was motivated by an array of political, social and economic factors unique to their lives.

The mainstream opinion in this sample of respondents was a great appreciation that the trial provided free, high‐quality medical care for their child during difficult economic conditions.

R40: Before the research study, when you visit the government hospital after tests they were telling you to go buy medicines and sometimes you do not have the money. But after my child joined the study, the situation changed. I am grateful my child was getting malaria tests and given medicines in a sealed bottle not the opened ones.

The contrast between the free medical care in the study and the options available to the parents through government hospitals was consistently highlighted by respondents.

R56: We were comfortable because the health workers were approaching us well, they had good manners. At times, you may go to the hospital and they tell you that you are stupid, but these ones were good health workers, they could tell you to do something and you accept because of their approach.

The trial was also not independent from circulating rumours within social networks. Where the mainstream in this sample reported an appreciation for the trial and the medical benefits, a few outliers noted the impact rumours had on the way the study was perceived and pushed back against them.

R34: Someone can spread rumours. We would tell them to go and see for themselves that there is nothing negative taking place. My child who is in the study is healthier than yours who is not but you keep talking about blood draws. You destroy the image of the study for nothing.

### Community context

The community played an integral role in the uptake, acceptability and integration of the clinical trial into the local setting. Caregiver decision‐making was intimately tied to their relationship with others in their local community.

R46: Before joining, I used to see my friends going and I guessed it would be a good project. When I joined, I was assured, yes, it was a good project based on their procedures and services given.

A number of participants reported that the benefits of the trial should be available to everyone. Placing an emphasis on the need for the high standard of health care to be extended towards other members in the community.

R32: Your neighbour ought to enjoy what you enjoy. The fruits you enjoy, he ought to enjoy.

Respondents often reported from the perspective of the community and how the trial improved conditions for the children of their community as a whole, despite a lack of financial resources.

R35**:** The people were enrolled praised the study. Most of them are the people who come from around who earn a little money. Sometimes when the child falls sick it becomes difficult especially for us who are farmers. They would give the children effective drugs. So, the people around consider it good.

The local political leader has a significant influence on the trial. When the local leader is trusted by the community members and this individual approves of the trial, then the study participants will be much more comfortable.

R64: Our chairman as you have seen him, he is good. Whenever the study people would leave, he could explain to us what was going on, so that is how you could pick to participate. These ones who came straight to the chairman we knew that they are people of light, because we knew that someone who has not come through the chairman is the one you can doubt but someone who has come through the chairman, there is no need to question.

Where the mainstream in this sample reported satisfaction with the clinical trial, some outliers also reported cases where community members challenged the clinical trial and those enrolling their children.

R65: Because at first, the people were asking, why do you need our children? Which kind of check‐up are you doing? What are you checking? As you know the village life we are in.

### Domestic context

The caregivers of participants in the malaria vaccine clinical trial repeatedly spoke about the role that the trial played in their lives at home, in particular how the family's access to medication influenced their participation.

R14: My child she was very sick and when I went with her to the hospital I found the government sector had no drugs and the study did have drugs. So, I went and found a sister and she asked me if I could agree to join the study, ‘if you agree to join then I will take you so that your child can be helped and if you refuse, you can go to other district hospital but even there are no drugs.’ So, I sat with her and asked her and she had already told me that the study is very good. I asked her if they can help me and she said yes, only if I agree to join, and I said yes I have agreed to join and for sure they treated my child.

Facing financial challenges within the domestic settings and then having the clinical trial provide free and high‐quality care for the sick children was positive according to caregivers.

R66: I benefitted because my child is still alive.

R35: My child would be given medication even when I did not have cash. They would also give me fare back home. It was good. Anytime I would take the child to the hospital, they would treat him.

Fathers played a significant role in participation and many respondents who were mothers elucidated the role the father had in motivation and consent to join, or withdrawal from the study.

R17: Others also took it seriously that those people are removing a lot of blood from the children. So, they did not agree, and other people, including the fathers of the children never agreed which is why they did not join.

Caregivers also explained what it means for them to have a sick child within the home, particularly how it could also lead to problems within the relationships. For the majority of participants, this was improved when the children could participate in the study.

R56: You need to eat yet the child is sick. You eat late because food is prepared late, you quarrel and can even fight. Such things happen and there is no love in the family because every time you are concentrating on the child. You may find that even some men get other women, complaining that they are fed up of the other one because her children are sickly.

While the mainstream opinion in this qualitative sample expressed appreciation of the trial and the way in which it benefited the families, in some exceptional cases participation in the trial could lead to problems in the spousal relationship.

R78: They were removing a lot of blood. Maybe they just needed a lot. I was afraid that he will collapse and his father will beat me up. I was afraid but there were some women that we went with who encouraged me to go.

## Discussion

The findings of our qualitative in‐depth interviews with caregiver of participants enrolled in a paediatric malaria vaccine clinical trial provide insight into the values that caregivers hold, what motivates their participation, and their experience in the clinical trial. The primary motivation for participation drew from each theme (trial context, community context, domestic context) and is intricately connected to the political, social and economic reality that a caregiver occupies at a given time. Below, we move through these themes and discuss the role local values and beliefs play in research participation.

What is most striking about our results is the dominance of free medical care as being the prevailing motivator for participation. Limited capacity of local medical services has been raised as a challenge in transnational research when the medical services in the clinical trial significantly surpass local services 20, 21. It is cross‐cutting across all themes analysed in our study and is repeatedly emphasised by caregivers of participants enrolled in the clinical trial as being the most valued and positive component of the research trial. This finding illustrates the interplay between the local structural limitations and trial enrolment, having significant implications for individual decision‐making processes concerning the trial 22, 23. Having a powerful motivating factor, such as the provision of care in this context, prevalent across all themes, is indicative of local structural constraints. The provision of care to participants in clinical trial research is presented as ‘benefit‐sharing’ where the clinical trial aims to give back to participants. This ‘benefit‐sharing’ with individual participants in a setting with ill‐functioning institutional health care may impede choice with regards to enrolment 24. Failing to balance the provision of care with concise communication around trial proceedings to the caregiver can lead to an ‘empty choice’ where structural factors around health care eliminate an autonomous decision 25, 26. A caregiver of a participant will be limited in his or her autonomy when faced with the decision to enrol when it is the only means to ensure their child's health. This is relevant for both informed consent, but also risk *vs*. benefit communication in transnational clinical trials 20, 26, 27.

Beyond the individual, the provision of accessible medical care was also highlighted as the trial component most highly valued by the community. Health care was framed as a community value by respondents. The political leadership which influenced community acceptance of the research suggests a locus of decision‐making that is communal. The leadership decided its position on the research study and then passed this approval down into the community, driven by the desire to promote the health of the children. The provision of health care within the community context in combination with the structural constraints impacts decision‐making structures in clinical trials and provides challenges for the consent process 28. Communal decision‐making extends beyond traditional liberal political philosophical notions of autonomy and informed consent, this contextual reality was described by one caregiver as ‘*the village life we are in*’. The respondents are embedded in communal lives where other members of the community would suggest their child was going to be killed or face the consequence of a stigmatised condition if they enrolled in the malaria vaccine clinical trial. Failing to recognise the contingency of community and individuality and to overlook the historical experiences that contributed to the generation of these beliefs can derail research studies 29. Our results, in combination with the contingent notion of community in informed consent processes, place an emphasis on the need to clearly communicate risks and benefits to the trial community so that they are not overshadowed by the benefit associated with healthcare provision.

The final analysis of the domestic context also brought the value of healthcare provision to the forefront as the dominating motivator for trial participation. Having a sick child in the home leads to difficulties for others sharing that same domestic setting, whereas having access to health care to treat the sick child leads to greater domestic harmony. Respondents reported that enrolling in the research trial often occurred as a result of the difficulty in accessing medical care and trial enrolment has been reported to be lower in areas with better medical services 30. This illustrates the power that the provision of medical care has when it is embedded in a low‐resource context, distorting a balanced risk and benefit analysis or leading to the negation of the risks all together.

How parents weigh the risks and benefits of participation differed and was related to the structural constraints around healthcare access for the child 25. Health care provided by the clinical trial was highly valued by both parents and improved the condition of the child. This trickled down into having effects on the relationship the mother had with the father as well as the overall ‘*joy*’ in the home. Having a sick child in the home can burden the relationship and make parents more likely to participate in research than when their child is healthy 30. Individuals living in contexts with few medical services will see medical provision as a much larger benefit to their family than those in contexts with a strong local health system, calling for a tailored communication approach appropriate for each setting.

The absence of risk in the interviews conducted was also of note. Sceptical beliefs or concerns were often framed as ‘*rumours*’ by respondents and those believing them were referred to as a distant third party. Respondents repeatedly emphasised the gratitude they experienced from trial enrolment and the accompanying care. Concerns around potential adverse events outlined in the informed consent documents associated with vaccination did not come up as a significant concern during the interviews. While therapeutic misconception was also evident in some interviews, the offer of medical care overshadowed it in its ability to influence the caregiver's risk perception.

## Conclusion

Designing vaccine clinical trials in low‐resource settings such that the communication of risk and benefits is done in a way that is comprehended by participants and their communities is a challenging task. Ethical design of research requires the communication of trial proceedings not to be overshadowed by the provision of free care in resource‐limited settings. To address this in future trials and taking the first step towards more ethical communication means placing the community at the forefront of research design 31, 32, 33. Putting the community central to the research means to understand the values present in the settings where the research is taking place and how these are situated relative to the individual and their decision‐making processes. This can be achieved through stronger engagement with local stakeholders and health systems, including strengthening the government health system 34. The second step is to design clinical trials in collaborative partnership with local leadership to foster local capacity building and ultimately strengthen local health capacities 35. Being responsive to community needs and integrating values that influence participation in research alongside local leadership can provide a more balanced conception of the risks and benefits. The active involvement of both community and local leadership can support the disentanglement of comprehension barriers while still allowing for ‘benefit‐sharing’. An iterative process executed by the clinical trial team that engages the community and works closely with local leadership will foster research communication and thereby participant choice.

Understanding and addressing the local context will reduce inequalities inherent in transnational clinical trials in low‐resource settings 36. The utilisation of this understanding and its translation into research will support the communication of research appropriate for the local setting 37, 38. This involves integrating the social, political and economic components into clinical trial design and paving the way towards more equitable research practices and infrastructure that enables a real choice for study enrolment.

Limitations of this study include the sampling strategy, which recruited caregivers who enrolled their child and therefore would have been more likely to have a reduced risk perception due to the provision of care than caregivers who were approached and refused to enrol their child in the clinical trial. Future work investigating the perception of caregivers in the community who refused to enrol their child could shed further light on this topic. We also did not interview and male caregivers, which is indicative of the traditional caregiving roles where the mother or grandmother has the primary responsibility for the child's health.

Through mapping how contextual realities interplay with the decision‐making process of caregivers of paediatric clinical trial participants, this study can strengthen clinical trials in low‐resource settings. Our work shows that individual consent in clinical trials is intricately linked with community consent and family dynamics. Based on this, future research needs to investigate how this interplay varies across contexts and the role free medical care plays in consent in these settings.
